# Editorial: World no tobacco day 2023

**DOI:** 10.3389/fpubh.2025.1676372

**Published:** 2025-09-12

**Authors:** Sonu Goel, Garima Bhatt

**Affiliations:** ^1^Department of Community Medicine and School of Public Health, Post Graduate Institute of Medical Education and Research, Chandigarh, India; ^2^School of Medicine, Faculty of Education & Health Sciences, University of Limerick, Limerick, Ireland; ^3^Department of Health Sciences, University of York, York, United Kingdom

**Keywords:** tobacco control, food, FCTC, collection, environment

## Introduction

31 May marks World No Tobacco Day (WNTD), an annual global campaign led by the World Health Organization (WHO) to spotlight the dangers of tobacco use, expose the deceptive practices of the tobacco industry, empower people to assert their right to health and protect future generations from tobacco-related harm (1). For the year 2023, the theme for WNTD was “Grow food, not tobacco” pointing out the ways in which ensuring food security in the face of global change requires sustainable food production (2).

There is well-established evidence of the irreparable harm that tobacco causes to health (7 million deaths annually) (3), the environment (600 million trees are chopped down every year and 766,571 metric tons of cigarette butts, along with 894,700 e-cigarettes, are littered) (4) and farmers (economic hardships, labor exploitation, environmental degradation, and health problems) (5). Tobacco cultivation is resource-intensive and not only damages public health by fueling tobacco-related diseases but also leads to environmental degradation (e.g., deforestation, pesticide pollution, contaminating water sources, and soil depletion) that harms ecosystems and exacerbates climate change, thereby jeopardizing future food security (6). In the past decades, there has been a global shift in tobacco cultivation from high-income countries to low- and middle-income regions. This shift has contributed to environmental harm, including soil degradation and ecosystem disruption, driven by the heavy use of agrochemicals and deforestation for tobacco curing. These practices have also adversely affected the health of smallholder farmers, exposing them to hazardous chemicals and exacerbating food insecurity (7). Furthermore, tobacco production emits 80 million tons of carbon dioxide equivalent every year (8).

## Special collection on World No Tobacco Day

The Research Topic of nine articles in the current “Frontiers of Public Health” issue focuses on the 2023 WNTD theme “Grow food, not tobacco”. The contributions explored key public health and policy issues related to tobacco control, including the environmental impact of cigarette filters, exposure to secondhand smoke, the dual use of cigarettes and e-cigarettes, gender inequity and smoking patterns, public perceptions of e-cigarette regulations, compliance with tobacco vendor density laws, and industry interference in policymaking.

One article discussing the proposed ban on cellulose acetate cigarette filters in the European Union presented the key reasons for the ban, focusing on both human health and environmental impacts (Everaert et al.). It also examined the potential outcomes of such a ban and explored public opinion along with the tobacco industry's response. Two articles focused on secondhand smoke (SHS) exposure and smoke-free policies. One of these articles emphasized the need to develop targeted interventions to reduce SHS in homes and vehicles, particularly among youth belonging to racial, sexual, and gender minority groups (Talluri et al.). The tobacco industry markets smoking as a symbol of freedom and equality for women, especially younger ones, associating it with sophistication and fashion. This messaging makes women more likely to take up smoking or vaping as a form of empowerment or stress relief (9). The second article discussed factors influencing (positively and negatively) the adoption of comprehensive smoke-free policies by the local governments in cities of China (Feng et al.). The use of e-cigarettes as a replacement for smoking (displacement) or simply in addition to cigarettes (add-on use) was also explored in this Research Topic, highlighting patterns of dual use, its impact on smoking cessation, and public health implications (Kroeger et al.). The study suggests that dual use often fails to displace cigarette smoking and may undermine efforts to quit. Another article also described a two-way link between smoking and reduced quality of life, especially with regard to mental health, among teachers (Lizana et al.). Smokers were found to have significantly lower mental wellbeing scores, and those with poorer mental health were more likely to smoke. Gender-specific approaches were also found to be essential for effective tobacco prevention among youth. A time-based ecological analysis examined how changes in gender equality influenced smoking rates among 15–25-year-olds over 45-year-olds (Roczen et al.). As gender equality improved, smoking rates among young men and women became more similar, especially among those with a higher education. Another study analyzed U.S.-based Twitter/X public perceptions of the FDA's authorization of Vuse e-cigarettes (Lee et al.). It found that the majority of tweets were neutral, while negative posts which focused on health risks and criticized the decision, outnumbered positive ones. Although fewer, positive tweets often mentioned smoking cessation. Overall, the public discourse reflected more concern than support.

Reducing tobacco vendor density is an important measure for preventing tobacco uptake, especially among youth and for improving compliance with the law. A geospatial mapping study reported a high concentration of vendors, many violating tobacco control laws through sales to minors, advertising, and proximity to schools (Satpathy et al.). These findings highlight the urgent gaps in enforcement and the need for stronger policies. The tobacco industry has been using various tactics to influence health policy and increase its corporate social responsibility (CSR) efforts. One of the studies included here assessed India's efforts to reduce tobacco industry interference using the Global Tobacco Industry Interference Index (2019–2023) (Goel et al.). The findings report that while India initially improved its safeguards under WHO FCTC Article 5.3, that progress stalled in Goel et al.. The study calls for stronger regulations, greater transparency, and a unified government response.

Overall, these studies align well with the theme of the Research Topic, i.e., World No Tobacco Day, by emphasizing the need to curb the societal harms of tobacco and shift toward sustainable, health-centered alternatives.

## Conclusion and way forward

Given that tobacco cultivation poses a triple threat to health, the environment, and food security, a shift toward sustainable agriculture would be an essential step toward climate resiliency and sustainable development in addition to being a public health necessity. This shift could be supported by phasing out tobacco subsidies, fostering cross-sector collaboration between health, agriculture, and environmental sectors, and implementing the WHO FCTC Articles 17 and 18. This could be done by investing in the transition of farmers from tobacco cultivation to food production (10–12), tackling industry interference and countering the misleading narrative that tobacco farming leads to affluence (12). This approach is critical to achieving the Sustainable Development Goals (SDGs), particularly SDG 2 (Zero Hunger) and SDG 3 (Good Health and Wellbeing) (13).

## References

[1] World No Tobacco Day. Who.int. (2025). Available online at: https://www.who.int/campaigns/world-no-tobacco-day (Accessed July 25, 2025).

[2] World No Tobacco Day 2023. Who.int. (2023). Available online at: https://www.who.int/campaigns/world-no-tobacco-day/2023 (Accessed July 25, 2025).

[3] Tobacco. Who.int. (2025). Available online at: https://www.who.int/news-room/fact-sheets/detail/tobacco (Accessed July 25, 2025).

[4] Tobacco and the environment. Truth Initiative. (2019). Available online at: https://truthinitiative.org/research-resources/harmful-effects-tobacco/tobacco-and-environment (Accessed July 25, 2025).

[5] Tobacco Farming. Tobacco Tactics. (2020). Available online at: https://www.tobaccotactics.org/article/tobacco-farming/ (Accessed July 25, 2025).

[6] Novotny TE Bialous SA Burt L Curtis C Luiza da Costa V Iqtidar SU. The environmental and health impacts of tobacco agriculture, cigarette manufacture and consumption. Bull World Health Organ. (2015) 93:877–80. 10.2471/BLT.15.152744 26668440 PMC4669730

[7] Lecours N Almeida G. Abdallah JM, Novotny TE. Environmental health impacts of tobacco farming: a review of the literature. Tob Control. (2012) 21:191–6. 10.1136/tobaccocontrol-2011-050318 22345244

[8] Zafeiridou M Hopkinson NS Voulvoulis N. Cigarette smoking: an assessment of tobacco's global environmental footprint across its entire supply chain. Environ Sci Technol. (2018) 52:8087–94. 10.1021/acs.est.8b01533 29968460

[9] Goel S Walia D Kumar R. The hidden crisis: Health impacts of tobacco and nicotine products on Indian women. J Family Med Prim Care. (2024) 13:4751–4. 10.4103/jfmpc.jfmpc_1741_24 39723003 PMC11668366

[10] Tobacco Free Farms. Who.int. Available online at: https://www.who.int/initiatives/tobacco-free-farms/the-challenge (Accessed July 26, 2025).

[11] WHO Framework Convention on Tobacco Control & World Health Organization. WHO Framework Convention on Tobacco Control. Geneva: World Health Organization. (2003).

[12] Lencucha R Drope J Magati P Sahadewo GA. Tobacco farming: overcoming an understated impediment to comprehensive tobacco control. Tob Control. (2022) 31:308–12. 10.1136/tobaccocontrol-2021-056564 35241604

[13] Sustainable Development Goals. Undp.org. Available online at: https://www.undp.org/sustainable-development-goals (Accessed July 27, 2025).

